# Production of Lambic-like Fruit Sour Beer with *Lachancea thermotolerans*

**DOI:** 10.3390/antiox14070826

**Published:** 2025-07-04

**Authors:** Rubén Bartolomé, Elena Alonso, Antonio Morata, Carmen López

**Affiliations:** 1 Department of Chemistry and Food Technology, ETSIAAB, Universidad Politécnica de Madrid, 28040 Madrid, Spain; ruben.bartolome.perea@alumnos.upm.es (R.B.); elena.alonso@alumnos.upm.es (E.A.); 2 enotecUPM, Department of Chemistry and Food Technology, ETSIAAB, Universidad Politécnica de Madrid, 28040 Madrid, Spain; carmen.lopez@upm.es

**Keywords:** sour beer, *Lachancea thermotolerans*, fruit, non-*Saccharomyces*, antioxidant activity, volatile compounds, anthocyanins

## Abstract

Consumer demand for low-alcohol acidic beers is driving the use of non-conventional yeasts in the brewing process. In this study, the addition of mixed berries and fermentation with *L. thermotolerans* L31 are performed in crafting a low-alcohol acidic beer. Four different beers were brewed in the primary stage with either *Saccharomyces cerevisiae* or *L. thermotolerans* and with or without added berry mixture. Beer was fermented for 8 days at 20 °C, stored, and bottled. pH, density, alcoholic content, bitterness, and color of final beer were analyzed for all samples using analytical methods. Volatile compounds, anthocyanin content, and antioxidant activity were also evaluated. Sensory analysis was performed and correlated (PCA) with the analytical results. The obtained data indicated that beers brewed with *L. thermotolerans* were significantly more acidic and less bitter than *S. cerevisiae* beers. No difference in alcoholic content was found. Fruity aroma-associated compounds were present in *L. thermotolerans* beers, which correlated with the sensory analysis. Fruit beers were also redder and showed higher anthocyanin content and stronger antioxidant activity due to the presence of anthocyanins such as cyanidin, delphinidin, and malvidin from fruit, and other antioxidant compounds.

## 1. Introduction

Lately, the brewing industry has experienced an increase in the production of craft beers as an alternative to traditional brewing [1]. There are currently more than 150 different types of craft beer. Although the craft brewing process resembles many of the steps performed in traditional brewing, there may be some differences amongst them [2]. Due to its efficiency in ethanol production, its ability to metabolize maltose [3], and its ethanol tolerance [1], *Saccharomyces cerevisiae* is the main yeast used in beer making [4]. However, other non-*Saccharomyces* yeasts are now being studied and beginning to be used to produce craft beers [5] with unique flavors and aromas [6].

Belgian lambic beer is a type of sour traditional beer characterized by a higher concentration of organic acids, resulting in low pH [7] and acidic taste [8]. Traditionally, Belgian lambic beers are obtained by spontaneous fermentation carried out by bacteria and yeast that naturally exist in oak barrels were the wort is left to mature. The global process can take up to several months [9] and it is only performed during winter months, among many other limitations [7]. Therefore, the brewing industry is seeking innovative methods that allow enhanced production under controlled conditions and reduced-time processes, including controlled mixed fermentations and use of lactic acid bacteria (BAL) and acetic acid bacteria (AAB) [7,10].

An interesting alternative is the use of special non-*Saccharomyces* yeasts [11] that have low fermentative capacity and high enzymatic activity related to the production of volatile acids [12] and metabolites. These yeasts can produce low-alcohol beers [13] while contributing sensory characteristics similar to those of sour beers [14]. Among these yeasts, *Lachancea thermotolerans*, *Torulaspora delbrueckii*, and *Metschnikowia pulcherrima* stand out [15].

*Lachancea thermotolerans* (formerly *Kluyveromyces thermotolerans*) is a yeast species from the genus *Lachancea* that is found in several ecological niches, notably grape must [16]. It is used in winemaking to lower pH via lactic acid production and, therefore, to enhance sensory properties [17]. This heterofermentative yeast converts sugars into ethanol and lactic acid [18], metabolizing glucose, fructose, and sucrose similarly to *Saccharomyces cerevisiae*, although it cannot ferment maltotriose [18,19]. It exhibits the Crabtree effect [20], favoring fermentation over respiration in aerobic, glucose-rich environments, leading to ethanol production over biomass growth [21,22]. It consumes more sugar than *S. cerevisiae* to produce the same ethanol yield [17] and can produce lactic acid, reaching 1–9 g/L depending on the strain [20,23]. It is also appreciated for its ability to produce more glycerol than other yeasts [15,17]. Its growth is not significantly inhibited by hops or ethanol (up to 10% *v*/*v*) [24,25]. Additionally, it produces volatiles like 2-phenylethanol, ethyl lactate, and fruity esters, contributing to sweet and fruity aromas that are desirable in beers. These traits make *L. thermotolerans* an appealing starter culture for craft sour beer production [15,26,27].

In this study, *Lachancea thermotolerans* was used as a non-*Saccharomyces* yeast to produce a sour fruit beer by substituting bacterial acidification with yeast acidification, obtaining a final product with sensorial characteristics similar to those of lambic beers. This combination of traditional and modern methods in craft beer brewing opens new possibilities for the creation of differentiated, high-quality products.

## 2. Materials and Methods

### 2.1. Preparation of the Wort

The mashing process was carried out in a 40 L mash tun (Grainfather, Auckland, New Zealand), to which was added 6 kg of dehydrated and milled Château Pilsen 2RS malt (Castle Malting, Beloeil, Belgium), 37.5 g of roasted Carafa Type 1 malt (Weyermann, Bamberg, Germany) ground with a manual disc mill, and 18 L of tap water previously boiled and conditioned with calcium sulphate di-hydrate (CaSO_4_-2H_2_O) to a concentration of 90 mg/L Ca. The water was heated to 72 °C and added to the mash tun, and the mash tun was controlled to maintain a constant temperature of 68 °C for 60 min. The wort was washed in the same mash tun by lifting the inner lauter tun and adding 16.5 L of water previously adjusted to a calcium concentration of 90 mg/L with calcium sulphate dihydrate, heated to 77 °C, and added in three batches, two of 6.5 L and one of 4.5 L, allowing the water to drain between each batch. Once the wort had been washed, the bagasse was removed and the boiling process began, raising the temperature of the wort to 100 °C and boiling for 75 min. After the first 15 min of boiling, 30 g of Lemondrop hop pellets was added. After boiling, the wort was stirred and left to settle for 25 min.

### 2.2. Yeast Strains and Culture Media

Two yeasts strains, *Saccharomyces cerevisiae* 7VA and *Lachancea thermotolerans* L31 (Blizz™, Lallemand, https://www.lallemandwine.com/es/spain/productos/levaduras-enologicas/blizz/ (accessed on 1 May 2025)), were used for the first fermentation of the must. The yeasts were isolated in Ribera de Duero, Valladolid, and preserved by the microbiology department (ETSIAAB, UPM, Madrid, Spain) in tubes in YPD medium (1% yeast extract, 2% peptone, and 2% glucose) and 1.5% agar in the freezer. These strains were selected for several reasons. *S. cerevisiae* 7VA was selected for his high fermentative capacity, suitable fermentative kinetics, and low production of volatile acids. *L. thermotolerans* L31 was selected for its capacity to produce lactic acid. An initial growth was then carried out on the same medium in Petri dishes. The two yeasts were then inoculated in previously sterilized YPD liquid medium prepared for the growth of both yeasts separately. The inoculum was left in an oven at 28 °C for two days to promote growth.

### 2.3. Fermentation and Storage

The must at 20 °C was pumped into 12 different 2 L bottles to be used as fermenters, which had previously been autoclaved at 121 °C for 15 min, to a volume of 1700 mL each. Fermentations were carried out in triplicates. Berries were added to 6 of the 12 bottles, weighing 195 g of a mixture of Hacendado (Valencia, Spain) brand berries produced by Congelados de Navarra (Fustiñana, Navarra, Spain), containing strawberries, redcurrants, raspberries, blackberries, blackcurrants, and blueberries. The bottles were inoculated with 34 mL of yeast culture, corresponding to 2% of the final volume. The yeast inoculum concentration was 8 log CFU/mL. Six of the bottles were inoculated with *S. cerevisiae* and the other six with *L. thermotolerans*. Each bottle was fitted with a fermentation stopper that allowed CO_2_ from the fermentation to escape but did not allow microbial contaminants to enter the fermenter.

The fermenters were placed in an oven to keep them at a constant temperature of 20 °C for 8 days, with density, pH, and glucose samples taken every two days to monitor the fermentation, which was stopped when the density of the samples reached a value of around 1012 kg/m^3^.

Beers inoculated with L31 without added fruit did not complete fermentation and their density stalled above the target density. To complete the fermentation, they were re-inoculated with 0.4 g of yeast per liter of beer, using *S. cerevisiae* QA23™ active dry yeast (Lallemand Iberia, Madrid, Spain).

The final fermented beers were transferred to a cold room at 4 °C and stored for 7 days. The content of each bottle was then transferred to a clean, sterile bottle and re-transferred to clean bottles every two days to remove the solids and yeast residues. Table 1 shows the key to the experiment, indicating the characteristics, fermentation parameters, and number or replicates of each beer.

### 2.4. Bottling and Secondary Fermentation

The beer was transferred to sterilized 330 mL amber bottles. In order to produce CO_2_ in the beer, a secondary fermentation was performed. To achieve this, 0.4 g/L of active dry yeast *S. cerevisiae* QA23™ (Lallemand Iberia, Madrid, Spain) was added to each bottle. Sucrose (Hacendado, Spain) was dissolved in water and added to each bottle to a final concentration of 5 g/L. Finally, the bottles were capped with crown corks using a manual capper and the beers were stored in a cold room at 4 °C for 15 days for secondary fermentation.

### 2.5. Instrumental Analysis

#### 2.5.1. Density

The densities of both the wort and finished beer were measured at 20 °C in a 50 mL cylinder using densimeters in the ranges of 1000 to 1050 kg/m^3^ and 1050 to 1100 kg/m^3^, respectively.

#### 2.5.2. pH

All wort and finished beer samples were measured at 20 °C using a pre-calibrated Crison micropH 2000 pH meter (Barcelona, Spain).

#### 2.5.3. Glucose

Analysis of glucose concentration was carried out using the Food Quality Enology enzyme kit (Biosystems, Barcelona, Spain) by measuring the absorbance of the samples at a wavelength of 500 nm using a J.P. SELECTA UV-2005 UV–Visible spectrophotometer (Barcelona, Spain).

#### 2.5.4. Color

The color of the beer was measured spectrophotometrically using a J.P. SELECTA UV-2005 UV–Visible spectrophotometer (Barcelona, Spain), according to ASBC protocol number 10 [28]. The samples were filtered through a mixed cellulose ester (MCE) syringe filter with a pore size of 0.45 μm, and their absorbance was measured at 700 nm. The color was calculated in two different ways: the SRM and EBC scales [29].

#### 2.5.5. Alcoholic Content

The ethanol concentration of each sample was determined using combined methods of distillation and densimetry. First, 200 mL of each beer was distilled with 7 mL of lime slurry to neutralize it, and 100 mL of distilled water was added. The distilled hydroalcoholic solution was collected in a receiving flask until it was two-thirds full. The flask was made up to 200 mL with distilled water and transferred to a 250 mL graduated cylinder, wherein the temperature and alcohol content were measured using an alcoholmeter. As the temperature of the samples was not 20 °C, a correction was made using the correlation table of the International Organization of Vine and Wine (OIV).

#### 2.5.6. Bitterness

Bitterness analysis was carried out using a spectrophotometric method with a J.P. SELECTA UV-2005 UV–Visible spectrophotometer (Barcelona, Spain) by determining the α-acid concentration according to ASBC protocol number 23A [30]. First, 10 mL of each beer, 1 mL of 3M HCl, 20 mL of isooctane, and 50 µL of octanol were mixed in a 50 mL tube. The samples were vortexed and centrifuged to ensure complete separation of the phases, and the absorbance was measured at a wavelength of 275 nm using a 1 cm quartz cuvette. Bitterness was calculated in IBUs (International Bitterness Units) from the absorbance.

#### 2.5.7. Determination of Volatile Compounds

An Agilent Technologies 6850 gas chromatograph (GC System Network) equipped with a flame ionization detector (Hewlett-Packard, Palo Alto, CA, USA) was used to determine the concentration of the different volatile compounds present in the beers. A DB-624 column was used. The column temperature was 40 °C for the first 5 min, which was then increased by 10 °C per minute until 250 °C was reached. The injector temperature was 250 °C and the detector temperature was 300 °C. Hydrogen was used as the carrier gas. The method used to analyze the volatile compounds in the sample corresponded to the OIV protocol of volatile compounds (Resolution OIV-OENO 553-2016, https://www.oiv.int/public/medias/4968/oiv-oeno-553-2016-es.pdf, accessed on 20 January 2025).

The method used was calibrated by elaborating calibration curves of the following compounds in standard solutions: acetaldehyde, methanol, 1-propanol, diacetyl, ethyl acetate, 2-butanol, 2-butanol, isobutyl alcohol, 1-butanol, acetoin, 2-methyl-1butanol, 3-methyl-1-butanol, isobutyl acetate, ethyl butyrate, ethyl lactate, 2,3-butanediol, isoamyl acetate, hexanol, 2-phenylethanol, and 2-phenylethyl acetate (r^2^ > 0.999 for all compounds, LOD = 0.1 mg/L). Calibration curves were obtained in the form of hydroalcoholic solutions, with 13% *v*/*v* ethanol in water and different concentrations of between 1.000 and 500.000 mg/L of the compounds. 4-Methyl-2-pentanol (50 mg/L; Fluka Chemie GmbH, Buchs, Switzerland) was used as an internal standard (100 µL per sample).

Samples were evaluated in triplicate in the following order: 7VA, L31, 7VAF, and L31F. The column was washed with water between different types of beer to avoid interferences and instrumental soiling. Before measuring, samples were filtered through a 0.45 μm pore mixed cellulose ester (MCE) syringe filter, and 1 mL of the filtered sample was added to 1.5 mL chromatographic vials. The injection volume was 1 µL. The results were obtained in the form of chromatograms, in which the integration of each signal was performed to determine the concentration of each compound.

#### 2.5.8. Determination of Antioxidant Activity

The antioxidant activity of beer was determined using the ABTS method, as described in Re et al. (1999) [31]. Production of ABTS˙^+^ was obtained through the reaction of 7 mM ABTS and 2.45 mM potassium persulfate at room temperature for 24 h in the dark. Additionally, 2.5 mM Trolox (Sigma Aldrich, Merck, Burlington, MA, USA) was used as a standard. Next, 1 mL of diluted ABTS˙^+^ (A_734nm_ = 0.712) was added to 10 µL of sample/Trolox standard and left in the dark for 4 min. Then, absorbance at 734 nm was measured and used to calculate the antioxidant activity of samples in terms of Trolox equivalent antioxidant activity. Samples were analyzed in triplicate.

#### 2.5.9. Analysis of Anthocyanins Using HPLC-DAD

For the identification of anthocyanins, an Agilent Technologies (Palo Alto, CA, USA) series 1260 HPLC chromatograph equipped with a diode array detector (DAD) was used. The mobile phase consisted of a dilution of water/formic acid (95:5 *v*/*v*) (solvent A) and methanol/formic acid (95:5 *v*/*v*) (solvent B). The gradient was 0–2 min, 25% B; 2–10 min, 25–50% B linear; 10–11 min, 50% B; 11–12 min, 50–2% B linear; and 12–17 min, re-equilibration. The flow rate was set at 1 mL/min and the pressure of the column was higher than 200 bar according to the gradient composition. The temperature was 25 °C. The column used for the separation of pigments was a reverse-phase Poroshell 120 C18 column (Phenomenex, Torrance, CA, USA) with dimensions of 50 mm × 4.6 mm and a particle size 2.7 μm. The monitoring wavelength was set at 525 nm for the identification of anthocyanins. Samples were defrosted and filtered using 0.45 µm syringe filters. The volume of injection was 50 µL. Concentrations were calculated using a calibration curve with malvidin-3-O-glucoside as the external standard (*r*^2^ = 0.9999, LOD = 0.1 mg/L)

### 2.6. Sensory Analysis

Sensory evaluation was carried out with a panel of 10 trained judges from the Department of Chemistry and Food Technology of ETSIAAB (UPM). Each panelist tasted four beers, corresponding to the four different beer formulations. They rated 20 descriptive attributes using a tasting sheet: visual, olfactory, taste, aftertaste, and general perception. The attributes were rated on a five-point scale, with (1) low perception and (5) high perception. Tasting was carried out in a special room with white light, a controlled temperature of 21 °C, ventilation, and a sample preparation room.

### 2.7. Statistical Analysis

Determination was carried out in triplicate. Data were processed using Statgraphics v.16.2.04 (Graphics Software System, Rockville, MD, USA) and Microsoft Excel (2505 version). Two-way Analysis of Variance (ANOVA, LSD test) was performed considering two factors: yeast species (7VA, L31) and presence of berries (YES/NO). The level of significance was set at *p*-value < 0.05. In addition, XLSTAT version 1429 (2025.1.1) (Addinsoft, Paris, France) software was used to run a PCA and Pearson’s correlation test between the data obtained from the volatile analysis and the sensory test.

## 3. Results

### 3.1. Fermentation

**Figure 1:**
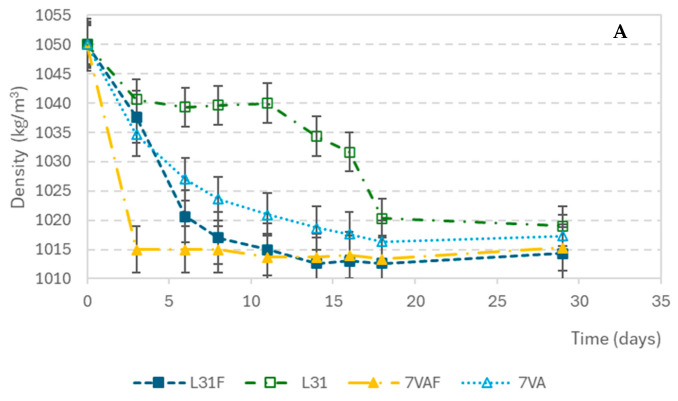

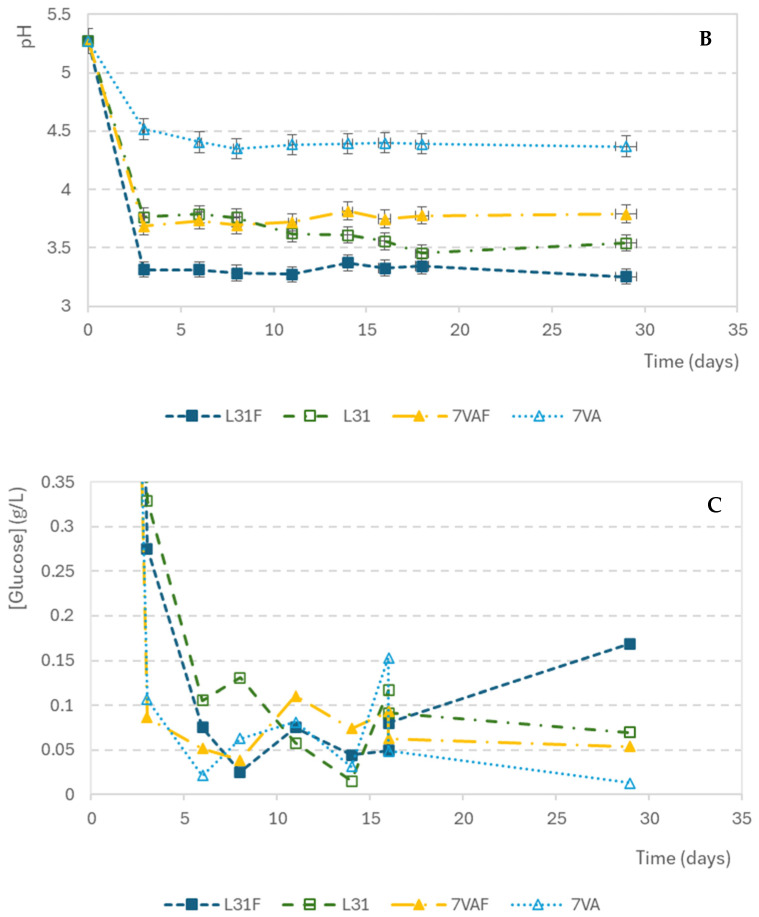
Evolution of parameters during fermentation: (**A**) Density (kg/m^3^); (**B**) pH; (**C**) glucose concentration (g/L).

The density variations during fermentation are shown in Figure 1A. The initial density of the wort was 1050 kg/m^3^ and it decreased progressively in all samples until it stabilized at around 1012 kg/m^3^ at the end of fermentation. The beers with the fastest decrease in density were those inoculated with *S. cerevisiae* and fruit, followed by the ones inoculated with *L. thermotolerans* with fruit, indicating that beers with fruit present the greatest fermentation rate. Something similar seemed to happen with pH variation. The pH decreased from the initial value of 5.27 during the first three days and then remained constant. L31F showed the fastest acidification rate (Figure 1B). As for the glucose concentration drop, shown in Figure 1C, it showed a high consumption rate in the first days, from an initial concentration of 2.83 g/L to values below the sensitivity of the measuring method (0.0023 g/L).

The finished beer analyses showed that both the type of yeast used and the addition of berries had significant effects on several of the measured parameters. The results are presented in Table 2.

Beers brewed with *S. cerevisiae* were 43.77% more bitter than those fermented with *L. thermotolerans*. The difference in bitterness may have been due to the influence of the yeast used. In addition, beers without red berries were 53.9% more bitter than beers with berries. Beers brewed with L31 were more acidic, with the pH ranging from 3.3 to 3.6, compared to beers brewed with 7VA, with values between 3.7 and 4.4. The type of yeast used also influenced this parameter, since significant differences could be appreciated between beers inoculated with L31 or 7VA. Also, beers with berries were significantly redder than those without berries. No influence of L31 or 7VA on the final color could be proven.

No significant differences were found in glucose concentration or final density. The residual glucose levels in the final beers were very low, indicating that both yeast strains consumed almost all of the available glucose during fermentation. Although no significant differences were found, a slightly higher concentration of residual glucose could be appreciated amongst beers brewed with the same yeast. This was probably due to the contribution of the sugar in the fruit to the beers. The ethanol contents in the final product of the four beers were similar (3.79–4.37% *v*/*v*) and not dependent on the choice of yeast or whether berries were added during the fermentation.

### 3.2. Volatile Compounds by GC-FID

The results obtained from GC-FID analysis are summarized in Table 3.

Regarding alcohols, such as methanol and 1-propanol, their concentrations exceeded the perception threshold in all samples. On the other hand, neither 2-butanol nor 1-butanol were detected. The isobutanol concentration was below the perception threshold, which meant that it was not detectable in the sensory profile of the beers. 3-Methyl-1-butanol, related to wine and banana aromas, exceeded the perception threshold only in sample L31, while 2-methyl-1-butanol, associated with similar aromas, showed concentrations above the perception threshold in all beers. Hexanol, related to herbaceous odors, showed concentrations above the perception threshold in beers fermented with *L. thermotolerans*, while beers fermented with *S. cerevisiae* showed slightly lower levels. 2-Phenylethyl alcohol, known to contribute to rose petal and perfume aromas, was present in all beers at levels above the detection threshold.

Levels of fruity aroma esters such as ethyl acetate and isobutyl acetate were higher in *L. thermotolerans* beers. Ethyl butyrate was not detected in any of the samples, while ethyl lactate was present at concentrations below the perception threshold. Isoamyl acetate and phenylethyl were found in all beers at concentrations above the perception threshold. These compounds contribute to aromas such as honey, rose, and apple.

Among carbonyl compounds, acetaldehyde, diacetyl, and acetoin were all present at concentrations over the perception threshold. These are responsible for apple, buttery, and woody aromas, respectively.

### 3.3. Sensory Analysis

In terms of visual attributes, beers with no berries (7VA and L31) showed goldish colors, this last one being darker. The addition of fruit significantly influenced both color and turbidity, resulting in red and hazy beers due to suspended fruit particles. Beers without fruit showed higher foam consistency and persistence.

Concerning the olfactory profile, the addition of fruit significantly affected the intensity and aromatic quality. Beers with no berries had higher aromatic intensity, with 7VA considered to be the best amongst the jury. In beer with berries, the aroma intensity was lower and fruitier, while beers without berries exhibited floral and malt as the principal aromas.

Finally, speaking of flavor attributes, all beers were rated as medium to low bodied, although L31 and L31F seemed to be fuller bodied. Samples with berries added were sweeter and more acidic. L31F showed the highest score, correlating to acid production by *L. thermotolerans*. By contrast, beers without fruit showed higher bitterness and effervescence. Astringency was low and similar in all samples. The aftertaste was more pronounced in beers without fruit and those fermented with *L. thermotolerans*, especially in sample L31.

### 3.4. Correlation Between Volatile Compounds Identified by GC-FID and Sensory Analysis

**Figure 2:**
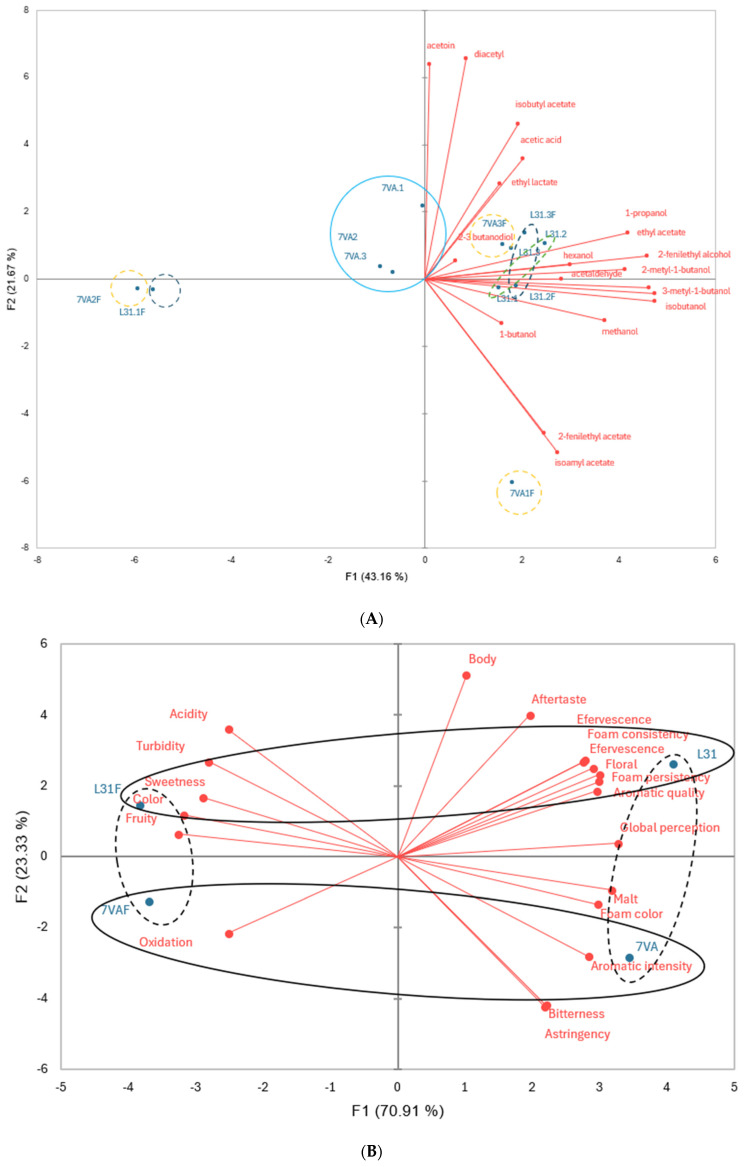
Principal component analysis of (**A**) volatile compounds in beers by GC-FID; (**B**) of sensory analysis. Different colors indicate different groups of beers.

For a better understanding of these results, principal component analysis (PCA) was performed and the results are shown in Figure 2. The first PCA, based on volatile compounds, identified seven groups of beers with distinct chemical profiles, while the second PCA, focusing on sensory analysis, reduced the number to two separated groups. The results clearly indicated differences in sensory attributes between beers depending on the presence of fruit and the yeast used during the fermentation step.

The correlation between the volatile compounds and the sensory analysis of the different beers was analyzed using Pearson’s correlation coefficient. The results are summarized in Table 4.

Positive correlations were found between aromatic intensity and compounds such as diacetyl, acetic acid, isobutyl acetate, and 2,3-butanediol. Aromatic quality also showed positive correlations with several volatile compounds, while floral aroma was related to many alcohols and esters present in samples analyzed by GC-FID. Contrary to expectations, the fruity aroma attribute did not show positive correlations with compounds typically associated with fruity aromas.

### 3.5. Anthocyanin Levels and Antioxidant Activity

Total anthocyanin content analysis, HPLC, and the ABTS essay were performed to determine the antioxidant activity of beer. The data are compiled in Table 5.

While beers 7VA, 7VAF, and L31 had similar antioxidant activities, beers fermented with *L. thermotolerans* and with additional berries seemed to have the strongest radical scavenging power. This may have been due to the presence of berries and related to the higher anthocyanin content in beer with berries. The highest anthocyanin concentration (9.42 ± 5.61 for 7VAF) did not match the beer with highest antioxidant activity (1.49 ± 0.38 for L31F), contrary to expectations.

According to Table 5, the anthocyanin content in beers with no berries could not be determined by HPLC-DAD since the concentration was below the limit of detection (LOD = 0.1 mg/L). Beers with berries did have anthocyanins, although the concentration was very low. 7VAF showed the highest anthocyanin content of all samples (9.42 ± 5.61) but had similar antioxidant activity levels to 7VA and L31, while these two had no fruit in their composition. Although the differences seemed to be significant for antioxidant activity (*p*-value < 0.05), we could not ensure that the increased antioxidant activity was caused by the addition of fruit, since the variation was minor and could be consequence of many other factors.

**Figure 3:**
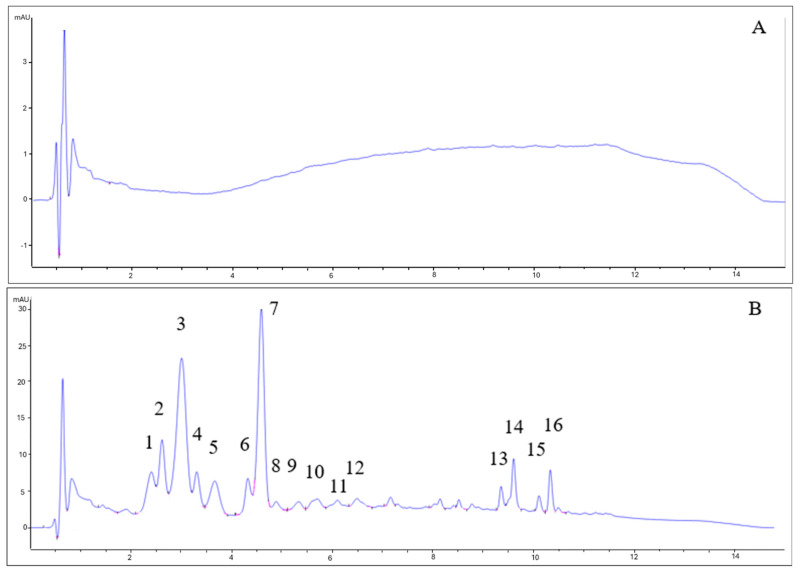

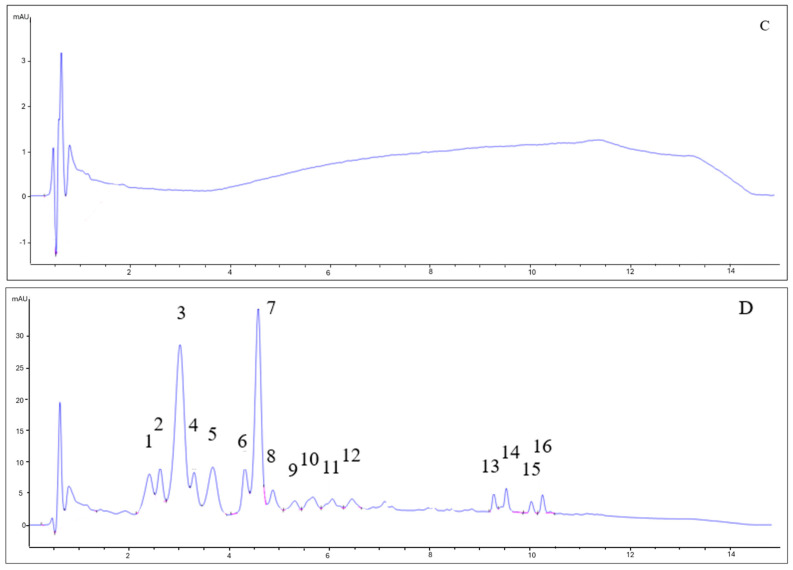
HPLC-DAD chromatograms of (**A**) 7VA, (**B**) 7VAF, (**C**) L31, and (**D**) L31F. Anthocyanins were measured at 525 nm. Numbers correspond to: delphinidin derivative (1), delphinidin-3-O-galactoside (2), delphinidin-3-O-glucoside (3), cyanidin derivative (4), pelargonidin derivative (5), cyanidin-3-O-glucoside (7), and malvidin-3-O-glucoside (11). Peaks 13–16 are apolar compounds.

As can be seen in Figure 3, the chromatograms for beers with berries and beers without berries showed completely different patterns. The type of yeast did not influence the anthocyanin profile. While beers without berries (7VA and L31) showed no remarkable peaks (all signals are lower than 1 mA), beers with berries had a very complex profile. Some of the identified peaks for fruit beer samples corresponded to delphinidin-3-O-galactoside (2) delphinidin-3-O-glucoside (3), cyanidin-3-O-glucoside (7), and malvidin-3-O-glucoside (11), by comparison with available data. Due to the low absorbance signal, weak UV–Vis spectra, and complexity, the rest of the peaks could not be properly identified, although peaks 1, 4, and 5 were thought to be delphinidin, cyanidin, and pelargonidin derivatives since these are the main anthocyanins in blueberries, blackcurrants, raspberries, and strawberries [33]. Peaks 13–16 were apolar compounds, probably beer aging products.

## 4. Discussion

This study evaluated the effect of different yeasts (*S. cerevisiae* and *L. thermotolerans*) as well as the addition of mixed berries in beer production and its significance in the physicochemical and sensory profiles of the final products.

During fermentation, the beers that lost density the fastest were those fermented with *S. cerevisiae* with fruit, followed by those fermented with *L. thermotolerans* with fruit, indicating that the samples with mixed berries fermented earlier and confirming the superior fermentative power of *S. cerevisiae* compared to *L. thermotolerans* [34,35,36]. Furthermore, during fermentation, *L. thermotolerans* showed a decrease in fermentative activity due to depletion of available sugars, while *S. cerevisiae* continued to ferment, possibly using other fermentable sugars remaining in the wort, such as maltose and maltotriose [19,37,38].

Beers fermented with *S. cerevisiae* were more bitter than those fermented with *L. thermotolerans*, suggesting that the yeast strain influenced bitterness. According to some studies, the α-acid molecules from hops can bind to yeast cell walls, which are deposited on the bottom of the fermenter, thus reducing the bitterness of the beer [39]. In addition, beers without berries were more bitter, which was consistent with other reviewed studies [17,40]. As previously seen with the type of yeast used, the α-acids may adhere to the cell walls of the fruit, reducing the bitterness of this type of beer.

Beers fermented with *L. thermotolerans* were more acidic than those fermented with *S. cerevisiae*. This was caused by the production of lactic acid by *L. thermotolerans*, which lowers the pH [15,17,32]. The pH values obtained in the four beer configurations were also in the range found by Polshin et al. [41]. The darker red color of beer with berries agreed with results obtained by Ducruet et al. (2017) in their studies on the brewing of craft beer with the addition of berries. These authors observed the extraction of compounds from berries due to the high formation of melanoidins during the Maillard reaction in the boiling phase [42].

The alcohol content of the beers studied showed no significant differences between the different yeast strains used, contrary to other studies such as the study by Callejo et al. (2019), where the ethanol content of beer produced by *S. cerevisiae* was significantly higher than that of beer produced by *L. thermotolerans* [19].

As for the volatile compounds, many alcohols (2-methyl-1-butanol and hexanol) were found in concentrations above the threshold and similar to those found in other studies, such as in the study by Peces-Pérez et al. (2022) [32]. A wide variety of esters responsible for the olfactory profile of the final product were found in concentrations similar to those reported by Ocvirk et al. (2018) [43] and even higher than those reported by Pavsler and Buiatti (2009) [44]. Amongst them, we can highlight ethyl acetate, isobutyl acetate, and acetaldehyde. These results also correlated with some recent studies on the matter, such as those by Zapata et al. (2019) [45] and Postigo et al. (2023) [35]. Beers crafted with *L. thermotolerans* were associated with herbaceous and fruity aromas, similar to lambic-type beers. Beers with *S. cerevisiae* were noted for higher intensity and malt-like aromas. Data from the sensory analysis also showed that beer brewed with *L. thermotolerans* L31 and with berries added was more acidic, less bitter, redder, and presented higher aromatic quality due to floral and fruity aromas and therefore had more similarities with lambic beers than 7VA, 7VAF, and L31.

Regarding antioxidant activity, anthocyanins are water-soluble compounds and the anthocyanin level should have been higher in L31F since its alcoholic graduation was lower. However, the antioxidant activity was higher in 7VAF, where the anthocyanin level was slightly lower. This meant that there were other antioxidant compounds, distinct from anthocyanins, that were not detected by the HPLC method but exerted antioxidant activity in the ABTS assay. Some possibilities include phenolic acids, melanoidin, or flavonoids present in malt, hops, and berries [46,47]. The extraction of these compounds is favored by methanolic solvents [48] and, therefore, higher ethanol content may aid the process. Since 7VAF had a greater ethanolic content, this could be an explanation for what we observed in this experiment.

Other studies claim that the ABTS assay is not able to determine the total antioxidant activity of all compounds in samples. Other methods, like the DPPH assay, work similarly and show higher values when performing both tests. According to Sariburun et al. [48], some differences can be appreciated when running one or the other while comparing dark-colored fruits like blackcurrants or blackberries and light red fruits such as strawberries and raspberries.

Some of the identified anthocyanins here, like delphinidin-3-O-galactoside, delphinidin-3-O-glucoside, or cyanidin-3-O-glucoside, are representative anthocyanins present in berries such as blackberries, blackcurrants, raspberries, and strawberries [49,50,51]. The fact that they only appeared in beers with fruits indicated that they had been extracted from the mixed berries to the wort during fermentation, affecting the antioxidant activity and color of these beers.

## 5. Conclusions

Overall, the choice of yeast influenced the physicochemical and sensory parameters of the final beer by 23%, while the addition of berries had a more significant impact on sensory attributes at 71%. Although beers without berries were rated better overall, the addition of berries may provide added value and differentiation to appeal to a more targeted audience. The combined use of *L. thermotolerans* and fruit contributes to producing sour low-alcohol beers, with an intense fruity aromatic profile resembling those of sour lambic beers. Future research could explore the interactions between different yeast strains and the influence of different fruits on the organoleptic and nutritional profiles of beer, contributing to innovation and diversification of the beer market.

## Figures and Tables

**Table 1:** Primary fermentation parameters.

Code	Yeast	Added Fruit (Yes/No)	Replicates
7VA	*Saccharomyces cerevisiae* 7VA	No	3
7VAF	*Saccharomyces cerevisiae* 7VA	Yes	3
L31	*Lachanchea thermotolerans* L31	No	3
L31F	*Lachanchea thermotolerans* L31	Yes	3

**Table 2:** Results of finished beer analysis.

	Bitterness (IBUs)	Glucose (g/L)	Density (kg/m^3^)	pH	Color (EBC)	Alcohol (% *v*/*v*)
L31F	8.58 ± 0.10 ^a^	0.10 ± 0.00046 ^a^	1015.33 ± 0.58 ^a^	3.37 ± 0.03 ^a^	18.35 ± 0.81 ^a^	3.91 ± 0.15 ^b^
L31	11.47 ± 0.91 ^b^	0.08 ± 0.00039 ^a^	1013.33 ± 1.15 ^a^	3.59 ± 0.03 ^b^	13.65 ± 0.63 ^b^	4.37 ± 0.30 ^d^
7VAF	10.72 ± 0.81 ^b^	0.06 ± 0.00023 ^a^	1015.33 ± 0.58 ^a^	3.74 ± 0.01 ^c^	18.14 ± 0.46 ^a^	4.02 ± 0.16 ^c^
7VA	18.65 ± 1.13 ^c^	0.03 ± 0.00014 ^a^	1017 ± 1.00 ^a^	4.33 ± 0.02 ^d^	13.73 ± 0.26 ^b^	3.79 ± 0.12 ^a^

Data are mean ± standard deviation (*n* = 3). Data with different letters within each column are significantly different (*p* < 0.05). L31F: *Lachancea thermotolerans* with fruit; L31: *Lachancea thermotolerans* without fruit; 7VAF: *Saccharomyces cerevisiae* with fruit; 7VA: *Saccharomyces cerevisiae* without fruit.

**Table 3:** Concentrations of volatile compounds (mg/L) in finished beers. Determined by GC-FID analysis. Perception threshold of different compounds in beer correspond to those indicated by Pérez-Peces et al. (2022) [32].

Volatile Compound	Perception Threshold (mg/L)	Associated Odors	L31F	L31	7VAF	7VA
Alcohols						
1-Butanol	450	n.d.	n.d.	n.d.	n.d.	n.d.
1-Propanol	3.0–16.0	alcohol	11.1 ± 7.3 ^a^	23.5 ± 1.5 ^c^	11.5 ± 7.3 ^a^	14.4 ± 3.2 ^b^
2-Butanol	16.0	n.d.	n.d.	n.d.	n.d.	n.d.
2-Methyl-1-butanol	8.0–30.0	alcohol, vinous, banana	13.7 ± 9.4 ^a^	20.5 ± 0.2 ^d^	17.1 ± 12.4 ^c^	16.7 ± 1.0 ^b^
2-Phenylethyl alcohol	8.0–35.0	rose petal, bitter	13.2 ± 3.9 ^b^	20.9 ± 3.1 ^d^	15.3 ± 5.8 ^c^	12.8 ± 0.2 ^a^
3-Methyl-1-butanol	8.0–30.0	alcohol, vinous, banana	23.0 ± 18.0 ^a^	36.7 ± 2.1 ^d^	26.9 ± 21.4 ^c^	25.0 ± 1.1 ^b^
Hexanol	4	herbaceous	5.9 ± 4.0 ^c^	4.3 ± 0.3 ^b^	3.1 ± 2.7 ^a^	4.0 ± 0.4 ^b^
Isobutanol	100	alcohol	14.9 ± 10.5 ^a^	21.7 ± 1.2 ^d^	15.5 ± 11.0 ^c^	14.3 ± 0.2 ^b^
Methanol	0.5–3.0	alcohol	19.6 ± 14.5 ^c^	15.3 ± 7.2 ^b^	19.0 ± 14.0 ^c^	13.9 ± 0.7 ^a^
Esters						
2-Phenylethyl acetate	0.05–2.0	roses, honey, apple	5.9 ± 1.2 ^a^	67.0 ± 0.5 ^b^	7.9 ± 4.2 ^c^	7.9 ± 0.5 ^c^
Ethyl acetate	30	fruity	20.3 ± 16.9 ^c^	34.7 ± 2.5 ^d^	17.6 ± 14.7 ^b^	16.6 ± 0.8 ^a^
Ethyl butyrate	0.4	fruity, tropical	n.d.	n.d.	n.d.	n.d.
Ethyl lactate	1.54	sweet, fruity, buttery	13.4 ± 9.9 ^d^	5.7 ± 5.7 ^b^	2.3 ± 4.0 ^a^	9.4 ± 3.6 ^c^
Isoamyl acetate	0.5	banana	2.4 ± 1.0 ^b^	2.6 ± 0.3 ^c^	2.9 ± 1.8 ^d^	2.0 ± 0.2 ^a^
Isobutyl acetate	1.6	banana, sweet, fruit	2.1 ± 2.4 ^b^	2.6 ± 2.3 ^c^	1.2 ± 1.3 ^a^	3.0 ± 1.7 ^d^
Carbonyl compounds						
Acetaldehyde	2.0–20.0	apple, green leaves	3.0 ± 2.1 ^b^	7.8 ± 3.9 ^c^	2.4 ± 1.6 ^a^	3.1 ± 0.5 ^b^
Acetoin	1.0–10.0	fruity, musty, woody	5.2 ± 0.6 ^b^	5.5 ± 0.2 ^bc^	3.3 ± 2.9 ^a^	5.6 ± 0.5 ^c^
Diacetyl	0.01–0.4	butter	1.4 ± 0.2 ^b^	1.7 ± 0.2 ^c^	1.0 ± 0.9 ^a^	1.7 ± 0.4 ^c^

Data are mean ± standard deviation (*n* = 3). Data with different letters within each column are significantly different (*p* < 0.05). L31F: *Lachancea thermotolerans* with fruit; L31: *Lachancea thermotolerans* without fruit; 7VAF: *Saccharomyces cerevisiae* with fruit; 7VA: *Saccharomyces cerevisiae* without fruit. n.d.: not detected.

**Table 4:** Correlations (Pearson’s test) between volatile analysis and sensory analysis.

	Aromatic Intensity	Aromatic Quality	Malt	Floral	Fruit Tree
Acetaldehyde	0.219	0.771 *	0.491	0.901 *	−0.586
Methanol	−0.968 *	−0.801 *	−0.986 *	−0.775 *	0.988 *
1-Propanol	0.419	0.816 *	0.638	0.948 *	−0.736 *
Diacetyl	0.736 *	0.944 *	0.912 *	0.816 *	−0.850 *
Ethyl acetate	0.019	0.656	0.310	0.801 *	−0.411
Isobutanol	0.063	0.590	0.305	0.779 *	−0.433
Acetic acid	0.992 *	0.734 *	0.972 *	0.680	−0.955 *
1-Butanol	−0.173	−0.241	−0.206	−0.511	0.382
Acetoin	0.518	0.883 *	0.749 *	0.712 *	−0.657
3-Methyl-1-butanol	0.215	0.572	0.390	0.784 *	−0.534
2-Methyl-1-butanol	0.437	0.525	0.508	0.743 *	−0.657
Isobutyl acetate	0.769 *	0.908 *	0.918 *	0.762 *	−0.844 *
Ethyl lactate	−0.059	0.264	0.102	−0.005	0.066
2,3-Butanediol	0.945 *	0.857 *	0.999 *	0.800 *	−0.983 *
Isoamyl acetate	−0.623	−0.567	−0.672	−0.313	0.524
Hexanol	−0.355	0.266	−0.089	0.078	0.207
2-Phenylethyl alcohol	0.031	0.471	0.227	0.696	−0.376
2-Phenylethyl acetate	0.598	−0.133	0.309	−0.029	−0.378

* Pearson’s correlation coefficients indicating strong correlations are marked with an asterisk (*).

**Table 5:** Antioxidant activity (µmol Trolox Equivalent/mL) and anthocyanin content (mg/L) in finished beers.

Beer	Antioxidant Activity (µmol TE/mL)	Anthocyanin Content (mg/L)
7VA	0.81 ± 0.23 ^a^	n.d.
7VAF	1.03 ± 0.35 ^a^	9.24 ± 5.40 ^b^
L31	1.03 ± 0.36 ^a^	n.d.
L31F	1.49 ± 0.38 ^b^	6.70 ± 2.56 ^a,b^

n.d.: not detected.

## Data Availability

The original contributions presented in this study are included in the article. Further inquiries can be directed to the corresponding author(s).
